# Association between the hOGG1 Ser326Cys polymorphism and lung cancer susceptibility: a meta-analysis based on 22,475 subjects

**DOI:** 10.1186/1746-1596-8-144

**Published:** 2013-08-23

**Authors:** Zhaoguo Xu, Li Yu, Xiaoye Zhang

**Affiliations:** 1 Department of Oncology, Shengjing Hospital Affiliated to China Medical University, Shenyang 110003, China

**Keywords:** hOGG1, Polymorphism, Susceptibility, Lung cancer, Meta-analysis

## Abstract

**Objectives:**

The Ser326Cys polymorphism in the human 8-oxogunaine glycosylase (hOGG1) gene with lung cancer susceptibility had been investigated, but results were inconsistent and underpowered. The aim of this study was to conduct a meta-analysis assessing the association of hOGG1 Ser326Cys polymorphism with risk of lung cancer.

**Materials and methods:**

Relevant studies were identified through a search of MEDLINE, PubMed, Web of Science, EMBASE, and Chinese Biomedical Literature database (CBM) using terms “lung cancer”, “hOGG1” or “OGG1”, “polymorphism” or “variation” and the last search updated on May 1, 2013. In this meta-analysis, we assessed 30 published studies involving 22,475 subjects that investigated the association between the hOGG1 Ser326Cys polymorphism and lung cancer susceptibility.

**Results:**

Overall, the hOGG1 Ser326Cys polymorphism was not associated with lung cancer susceptibility in different genetic models (dominant model comparison: OR = 0.133; 95% CI = 0.111–0.161; P_heterogeneity_ = 0.000), and recessive model: OR = 0.543; 95% CI = 0.399–0.739; P_heterogeneity_ = 0.000). Similarly, in the stratified analyses by ethnicity, significantly increased risks were found among Asians for homozygote comparison (OR = 0.850; 95% CI = 0.732 0.986; P_heterogeneity_ = 0.064), and dominant model (OR = 0.160; 95% CI = 0.137–0.187; P_heterogeneity_ = 0.001), and Caucasians for dominant model (OR = 1.35; 95% CI = 1.03–1.77; P_heterogeneity_ = 0.015), and recessive model (OR = 1.35; 95% CI = 1.03–1.77; P_heterogeneity_ = 0.015). In population-based populations, marginally significant increased risks were found in dominant model (OR = 0.143; 95% CI = 0.111 0.184; P_heterogeneity_ = 0.000) and recessive model (OR = 0.429; 95% CI = 0.261–0.705; P_heterogeneity_ = 0.000). We also found a significant difference between hOGG1 Ser326Cys genotype and lung cancer susceptibility in studies with hospital-based controls for homozygote model (OR = 0.798; 95% CI = 0.649–0.982; P_heterogeneity_ = 0.007),dominant model (OR = 0.122; 95% CI = 0.091–0.163; P_heterogeneity_ = 0.000).

**Conclusion:**

Our data showed that the hOGG1 Ser326Cys polymorphism contributed to the risk of lung cancer.

**Virtual slides:**

The virtual slides for this article can be found here: http://www.diagnosticpathology.diagnomx.eu/vs/3842531131031605

## Introduction

Lung cancer has become one of the most common malignancies all over the world with an extremely low survival rate is one of the leading causes to contribut to cancer mortality [1]. A number of environmental and genetic risk factors for lung cancer have been identified, and it is caused primarily by tobacco smoke, as manifested by the fact that 80% to 90% of lung cancer patients are smokers, while “only” 10% to 15% of heavy smokers develop lung cancer, suggesting the existence of personal risk factors of genetic origin, which predispose a fraction of smokers to the disease [2-5]. DNA damage may lead to carcinogenesis through inactivation of tumor suppressor genes or activation of oncogenes [6,7], and recent studies have focused on the association between genetic polymorphisms in different genes and risk of lung cancer [8-10], and they were certified by different techniques [11].

The 8-oxoguanine lesion is one of themajor forms of oxidative DNA damage [12,13], and it can be removed from DNA by human 8-oxoguanine DNA N-glycosylase 1 (hOGG1) [14]. This glycosylase has been suggested as a possible suppressor of lung carcinogenesis in OGG1-knockout mice [15], and in a bacterial complementation assay system the hOGG1 Cys326 allele was postulated to reduce the activity of 8-oxoguanine lesion removal [16]. 8-Oxodeoxyguanosine, the most abundant lesion generated by oxidative stress from the environment and normal cellular metabolism, is highly mutagenic resulting in GC to TA transversion [17,18]. hOGG1 gene located on chromosome 3 encodes a DNA glycosylase/apurinic-apyrimidinic lyase that catalyzes the excision and removal of 8-hydroy-2-deoxyguanine adducts [19]. An approximately 2-fold increased risk of lung cancer associated with the Cys/Cys or Ser/Cys genotype of hOGG1 has been observed in many different ethnicity populations [20-22]. In the past years, the hOGG1 Ser326Cys polymorphism has attracted widespread attention. Previous epithio studies were performed to identify the association of Ser326Cys polymorphism with lung cancer risk [16,20-46]. However, the results remain inconclusive and inconsistent. Therefore, a meta-analysis was performed in our present study to further evaluate the association between hOGG1 Ser326Cys polymorphism and lung cancer risk, by which can facilitate this disease prevention and diagnosis.

## Materials and methods

### Publication search

A comprehensive search strategy was conducted towards the electronic databases including MEDLINE, PubMed, Web of Science, EMBASE, and Chinese Biomedical Literature database (CBM) using terms “lung cancer”, “hOGG1” or “OGG1”, “polymorphism” or “variation” and the last search updated on May 1, 2013. Among the studies retrieved, eligible ones were determined and their bibliographies were evaluated for other relevant publications. Review articles and bibliographies of other relevant studies identified were hand-searched to identify additional eligible studies. Only published studies with full text articles were included. When more than one of the same patient population was included in several publications, only the most recent or complete study was used in this meta-analysis.

### Inclusion criteria and exclusion criteria

The following inclusion criteria were used to select literatures for the meta-analysis: (1) Only the case–control studies were considered; (2) The paper should clearly describe lung cancer diagnoses and the sources of cases and controls; (3) The authors must offer the size of the sample, OR and their 95% CI or the information that can help infer the results in the papers (provided the number of individuals homozygous for Ser/Ser, Cys/Cys and heterozygous for Ser/Cys in lung cancer cases and controls). The exclusion criteria were: (1) none-case–control studies; (2) control population including malignant tumor patients; and (3) duplicated publications.

### Data extraction

Information was carefully extracted from all eligible publications independently by two authors according to the inclusion and exclusion criteria listed above. An agreement was reached by discussion between the two reviewers whenever there was a conflict. The following data were collected from each study: first author’s surname, year of publication, country, ethnicity, source of controls (Population-Based and Hospital-Based population), and numbers of cases and controls with the Ser/Ser, Ser/Cys, and Cys/Cys genotypes, respectively. Different ethnicity descents were categorized as Caucasian and Asian population. When studies included subjects of more than one ethnicity and were able to separate, data were extracted separately for each ethnic group. We did not define any minimum number of patients to include a study in our meta-analysis.

### Statistical analysis

Crude odds ratios (ORs) with 95% confidence intervals (CIs) were used to assess the strength of association between the hOGG1 Ser326Cys polymorphism and lung cancer risk according to the method of Woolf [47]. Homozygote model (Ser/Ser vs Cys/Cys), heterozygote model (Ser/Ser vs Ser/Cys, Ser/Cys VS Cys/Cys), dominant (Ser/Ser + Ser/Cys vs Cys/Cys), recessive model (Ser/Ser vs Ser/Cys + Cys/Cys) and Ser-allele compared Cys-allele model (Ser-allele vs Cys-allele) were estimated, respectively. Subgroup analyses were done by ethnicity and source of controls. Both fixed-effects model using the Mantel–Haenszel method [48] and random-effects model using the DerSimonian and Laird method [49] were used to pool the results.

Heterogeneity assumption was checked by the Chi-square-based Q-test [50]. A P-value greater than 0.10 for the Q-test indicates a lack of heterogeneity among studies, so the pooled OR estimate of the each study was calculated by the fixed-effects model. Otherwise, the random-effects model was used. The significance of the pooled OR was determined by the Z-test, and P < 0.05 was considered as statistically significant. One-way sensitivity analyses were performed to assess the stability of the results, namely, a single study in the meta-analysis was deleted each time to reflect the influence of the individual data set to the pooled OR. Begg’s funnel plots [51] and Egger’s regression method [52] were used to assess publication bias statistically (p < 0.05 was considered representative of statistically significantly publication bias). Hardy–Weinberg equilibrium in the control group was tested by the Chi-square test for goodness of fit, and a P-value of <0.05 was considered significant. All of the calculations were performed using STATA version 12.0 (Stata Corporation, College Station, TX).

## Results

### Study characteristics

**Figure 1:**
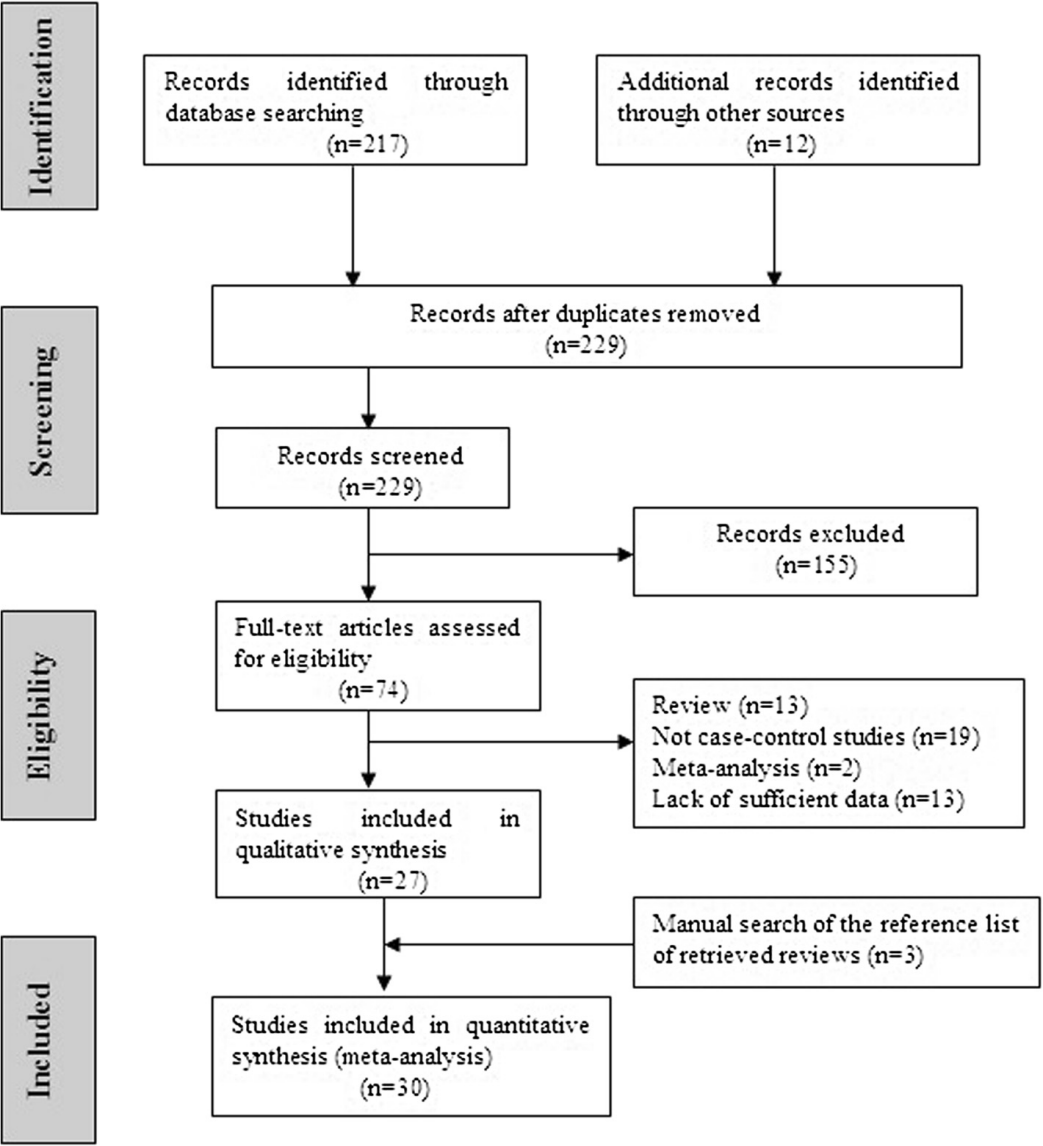
Flow chart illustrating the selection of studies.

Studies relevant to the searching words were retrieved originally. The initial search algorithm retrieved 229 references. After careful review of the abstracts, 155 of studies were excluded because they obviously did not meet the inclusion criteria for the meta-analysis. After this exclusion, 74 studies were left for full publication review. After review of the complete articles, 47 studies were excluded because of a lack of sufficient information or methods discrepancies. A total of 30 studies involving 10,327 lung cancer cases and 12,148 controls were ultimately analyzed (Figure 1). Table 1 presents the main characteristics of these studies. Almost all of the cases were histologically confirmed. Controls were mainly healthy populations. There were a total of 30 studies including 17 groups of Asians, and 13 groups of African-Americans. Simultaneously, there were 14 population-based studies and 16 hospital-based studies. The distribution of genotypes in the controls of all studies was in agreement with Hardy–Weinberg equilibrium except for three studies [27,37,44].

**Table 1:** Main characteristics of these studies included in this meta-analysis

**First author [Inference]**	**Year**	**Country**	**Ethnicity**	**Control source**	**Cases**			**Controls**			***P***_***HWE***_
					**Ser/Ser**	**Ser/Cys**	**Cys/Cys**	**Ser/Ser**	**Ser/Cys**	**Cys/Cys**	
Kohno	1998	Japan	Asian	PB	16	19	10	15	20	7	0.93
Le Marchand ^a^	2002	USA	Caucasian	PB	78	39	9	98	53	8	0.81
Le Marchand ^b^	2002	USA	Asian	PB	30	40	27	50	74	26	0.88
Lan	2004	China	Asian	PB	37	61	20	51	43	15	0.23
Vogel	2004	Denmark	Caucasian	PB	149	93	14	159	91	19	0.24
Loft	2006	Denmark	Caucasian	PB	144	93	14	154	88	19	0.20
Matullo	2006	Italy	Caucasian	PB	66	46	4	673	371	50	0.90
Sorensen	2006	Denmark	Caucasian	PB	254	155	22	479	284	33	0.26
Zienolddiny	2006	Italy	Caucasian	PB	182	100	44	194	117	75	**0.01**
Hatt	2008	Denmark	Caucasian	PB	92	58	8	93	59	12	0.54
Karahalil	2008	Turkey	Caucasian	PB	86	65	14	115	106	29	0.55
Okasaka	2009	Japan	Asian	PB	117	257	141	250	544	236	0.07
Li	2011	China	Asian	PB	83	208	164	60	219	164	0.33
Qian	2011	China	Asian	PB	100	288	193	125	291	185	0.59
Sugimura	1999	Japan	Asian	HB	85	115	41	63	107	27	0.08
Wikman	2000	Germany	Caucasian	HB	68	32	5	60	43	2	0.07
Ito	2002	Japan	Asian	HB	40	71	27	68	118	54	0.84
Sunaga	2002	Japan	Asian	HB	54	106	38	50	66	36	0.13
Park	2004	USA	Caucasian	HB	101	65	13	255	87	8	0.86
Hung	2005	European	Caucasian	HB	1401	661	93	1368	716	79	0.22
Liang	2005	China	Asian	HB	27	132	68	28	123	76	**0.04**
Wang	2005	China	Asian	HB	49	51	24	45	70	13	0.06
Kohno	2006	Japan	Asian	HB	285	544	268	123	190	81	0.63
De Ruyck	2007	Belgium	Caucasian	HB	74	33	3	60	46	4	0.18
Chang	2009	China	Asian	HB	142	518	436	154	482	361	0.74
Gao	2009	China	Asian	HB	27	35	24	30	49	11	0.19
Miyaishi	2009	Japan	Asian	HB	27	55	26	39	54	28	0.27
Liu	2010	China	Asian	HB	68	158	132	110	294	312	**0.01**
Janik	2011	Poland	Caucasian	HB	48	24	16	57	21	1	0.54
Kohno	2011	Japan	Asian	HB	115	162	100	98	164	63	0.70

^a,b^ Two different ethnicity studies in one publication. *PB* Population-Based Study, *HB* Hospital-Based Study, *P*_*HWE*_ P value of Hardy–Weinberg Equilibrium.

### Meta-analysis results

**Figure 2:**
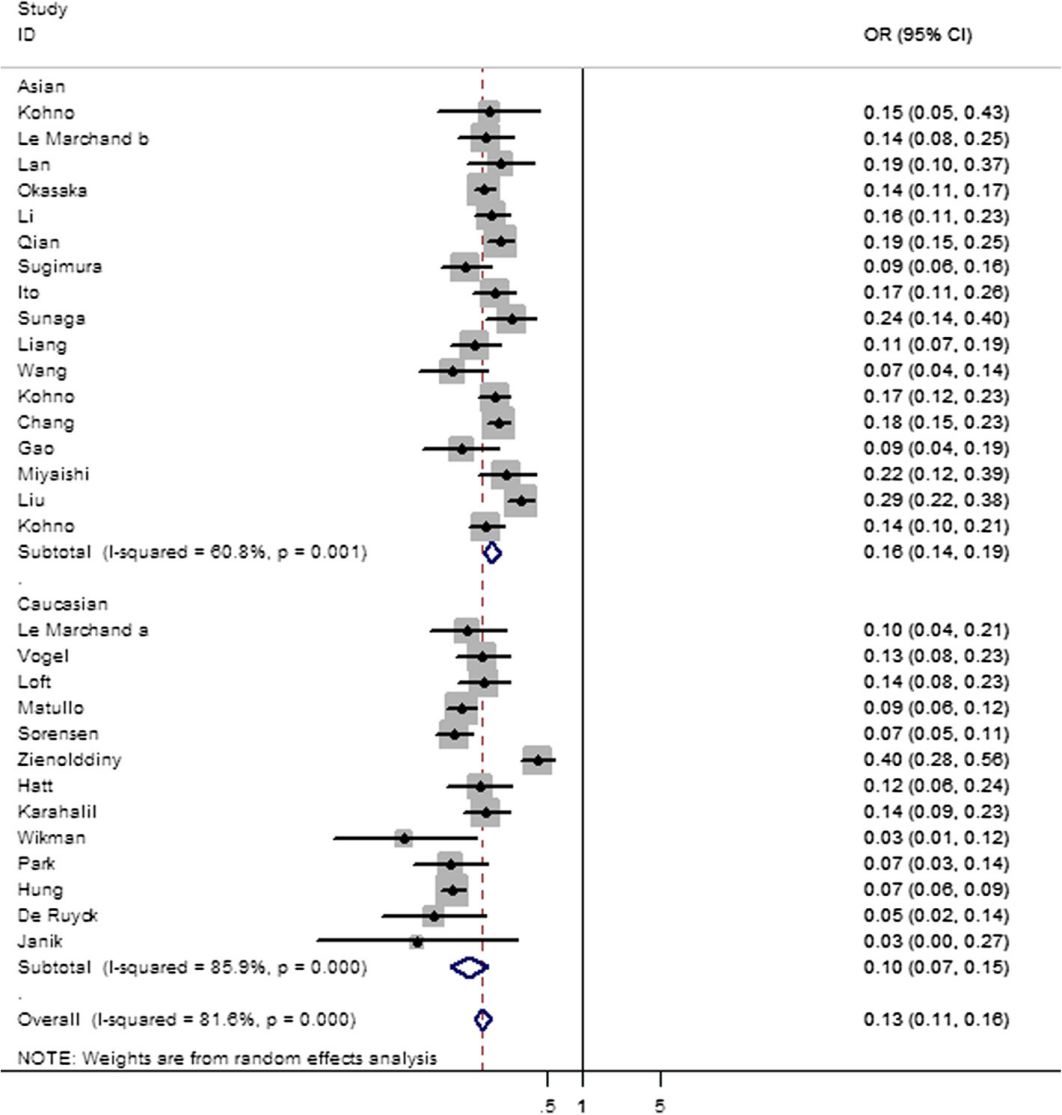
Forest plot of the odds ratios (ORs) and 95% confidence intervals (CIs) of studies of the association between the lung cancer risk and the hOGG1 Ser326Cys polymorphism (Dominant model comparison).

**Figure 3:**
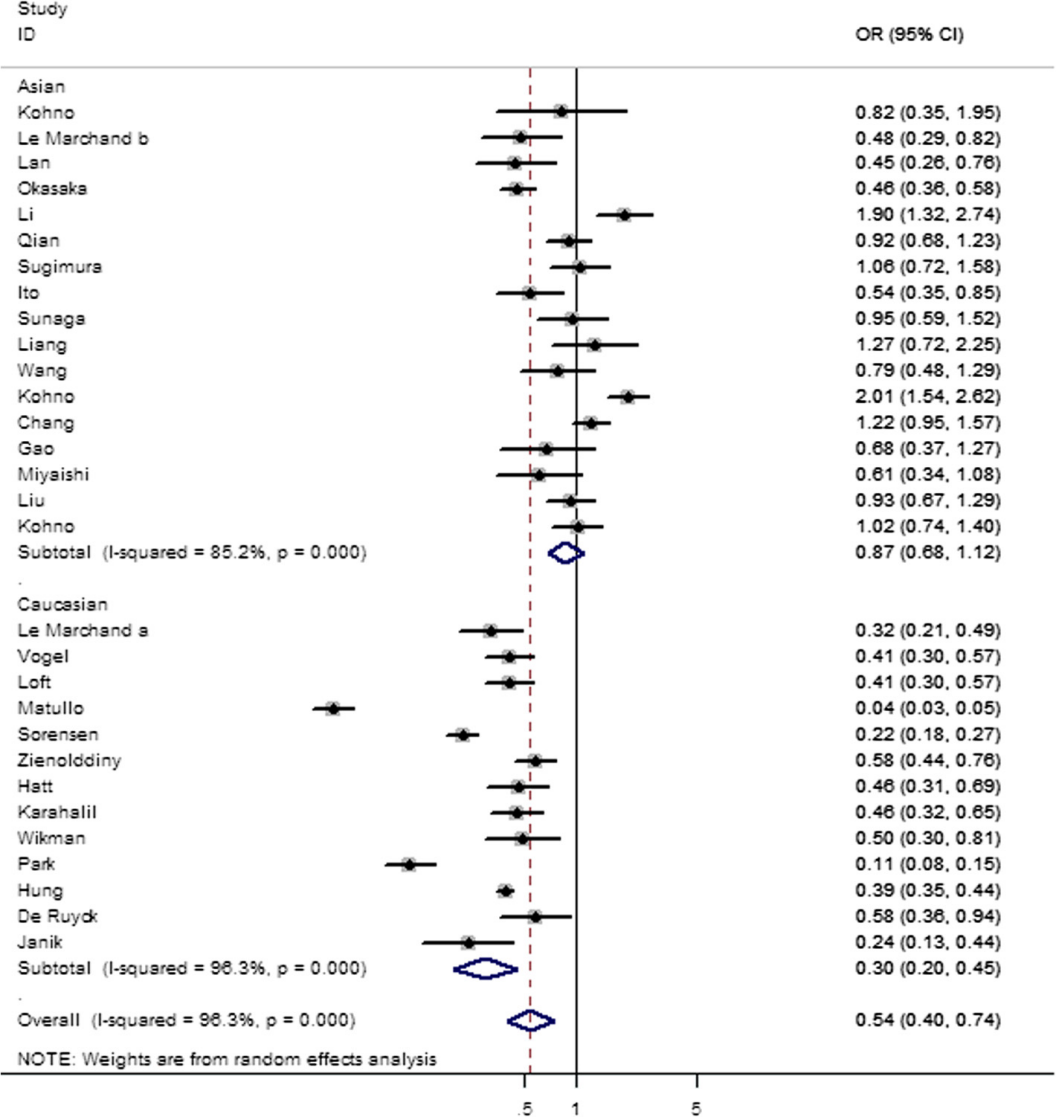
Forest plot of the odds ratios (ORs) and 95% confidence intervals (CIs) of studies of the association between the lung cancer risk and the hOGG1 Ser326Cys polymorphism (Recessive model comparison).

Table 2 lists the main results of this meta-analysis. Overall, significantly elevated lung cancer risk were associated in both dominant (OR = 0.133; 95% CI = 0.111–0.161; P_heterogeneity_ = 0.000, P = 0.000) (Figure 2), and recessive model (OR = 0.543; 95% CI = 0.399–0.739; P_heterogeneity_ = 0.000, P = 0.000) (Figure 3) when all the eligible studies were pooled into the meta-analysis. However, there were no significant associations in homozygote model (OR = 0.885; 95% CI = 0.765–1.024; P_heterogeneity_ = 0.002, P = 0.101), heterozygote model (OR = 0.982; 95% CI = 0.898–1.075; P_heterogeneity_ = 0.023, P = 0.698 for Ser/Ser vs Ser/Cys model; OR = 0.909; 95% CI = 0.802–1.029; P_heterogeneity_ = 0.006, P = 0.131 for Ser/Cys vs Cys/Cys), and also the Ser-allele were not associated with an increased cancer risk compared with the Cys-allele model (OR = 0.947; 95% CI = 0.880–1.019; P_heterogeneity_ = 0.000, P = 0.143).

**Table 2:** Main results of pooled odds ratios (ORs) with confidence interval (CI) in the meta-analysis

**Variables**	**No. of studies**	**Ser/Ser vs Cys/Cys**			**Ser/Ser vs Ser/Cys**			**Ser/Cys vs Cys/Cys**		
		**OR (95% CI)**	**P**_**h**_	**P**	**OR (95% CI)**	**P**_**h**_	**P**	**OR (95% CI)**	**P**_**h**_	**P**
**Total**	30	0.885(0.765 1.024)	0.002	0.101	0.982(0.898 1.075)	0.023	0.698	0.909(0.802 1.029)	0.006	0.131
**Ethnicity**										
Asian	17	0.850(0.732 0.986)	0.064	**0.032**	0.973(0.860 1.100)	0.102	0.659	0.887(0.771 1.020)	0.012	0.092
Caucasian	13	0.946(0.683 1.310)	0.005	0.738	0.993(0.866 1.138)	0.041	0.915	0.970(0.734 1.281)	0.070	0.829
**Source of controls**										
PB	14	0.993(0.810 1.216)	0.069	0.945	0.974(0.877 1.083)	0.377	0.630	0.956(0.833 1.098)	0.367	0.527
HB	16	0.798(0.649 0.982)	0.007	**0.033**	0.991(0.858 1.144)	0.007	0.900	0.837(0.689 1.017)	0.001	0.073
**Variables**	**No. of studies**	**Ser/Ser + Ser/Cys vs Cys/Cys (dominant)**			**Ser/Ser vs Ser/Cys + Cys/Cys (recessive)**			**Ser allele vs Cys allele**		
		**OR (95% CI)**	**P**_**h**_	**P**	**OR (95% CI)**	**P**_**h**_	**P**	**OR (95% CI)**	**P**_**h**_	**P**
**Total**	30	0.133(0.111 0.161)	0.000	**0.000**	0.543(0.399 0.739)	0.000	**0.000**	0.947(0.880 1.019)	0.000	0.143
**Ethnicity**										
Asian	17	0.160(0.137 0.187)	0.001	**0.000**	0.875(0.684 1.118)	0.000	0.285	0.931(0.865 1.000)	0.064	0.052
Caucasian	13	0.101(0.069 0.147)	0.000	**0.000**	0.300(0.198 0.454)	0.000	**0.000**	0.970(0.836 1.125)	0.000	0.686
**Source of controls**										
PB	14	0.143(0.111 0.184)	0.000	**0.000**	0.429(0.261 0.705)	0.000	**0.001**	0.979(0.897 1.069)	0.087	0.644
HB	16	0.122(0.091 0.163)	0.000	**0.000**	0.670(0.455 0.986)	0.000	**0.042**	0.916(0.817 1.026)	0.000	0.131

*P*_*h*_ P value of heterogeneity.

In the stratified analysis by ethnicity, significantly increased risks were found among Asians for homozygote model (OR = 0.850; 95% CI = 0.732 0.986; P_heterogeneity_ = 0.064, P = 0.032), dominant model (OR = 0.160; 95% CI = 0.137–0.187; P_heterogeneity_ = 0.001, P = 0.000) (Table 2 and Figure 2). Simultaneously, significantly increased risks were found among Caucasians for dominant model (OR = 1.35; 95% CI = 1.03–1.77; P_heterogeneity_ = 0.015) (Table 2 and Figure 2), and recessive model (OR = 1.35; 95% CI = 1.03–1.77; P_heterogeneity_ = 0.015) (Table 2 and Figure 3). However, no significant associations were found in both Asians and Caucasians for other genetic models (Table 2). When stratifying this meta-analysis by control sources, we also found a significant difference between hOGG1 Ser326Cys genotype and lung cancer susceptibility in studies with hospital-based controls for homozygote model (OR = 0.798; 95% CI = 0.649–0.982; P_heterogeneity_ = 0.007; P = 0.033), dominant model (OR = 0.122; 95% CI = 0.091–0.163; P_heterogeneity_ = 0.000; P = 0.000), and recessive model (OR = 0.670; 95% CI = 0.455–0.986; P_heterogeneity_ = 0.000; P = 0.042) (Table 2). In population-based studies, marginally significant increased risks were found in dominant model (OR = 0.143; 95% CI = 0.111 0.184; P_heterogeneity_ = 0.000; P = 0.000) and recessive model (OR = 0.429; 95%CI = 0.261–0.705; P_heterogeneity_ = 0.000; P = 0.001) (Table 2).

### Test of heterogeneity

There was significant heterogeneity for homozygote comparison (P = 0.002), heterozygote comparison (P = 0.023 for Ser/Ser vs Ser/Cys model and P = 0.006 for Ser/Cys vs Cys/Cys), dominant model comparison (P = 0.000), recessive model comparison (P = 0.000) and Ser-allele vs Cys-allele comparison (P = 0.000). After assessing the source of heterogeneity for all genetic model comparison by subgroup analysis on ethnicity and control sources, the heterogeneity was partly decreased. When we deleted these three studies [27,37,44] for departure from HWE, the heterogeneity was completely removed.

### Sensitivity analysis

A single study involved in the meta-analysis was deleted each time to reflect the influence of the individual data set to the pooled ORs, and the corresponding pooled ORs indicated that three studies were the main origin of heterogeneity. The heterogeneity was completely removed after exclusion of these studies. Although these studies did not follow HWE, the corresponding pooled ORs were not materially altered with or without including them almost in all genetic models. Similarly, no other single study influenced the pooled OR qualitatively, as indicated by sensitivity analysis, suggesting that the results of this meta-analysis are stable (Data were not show in this paper).

### Publication bias

**Figure 4:**
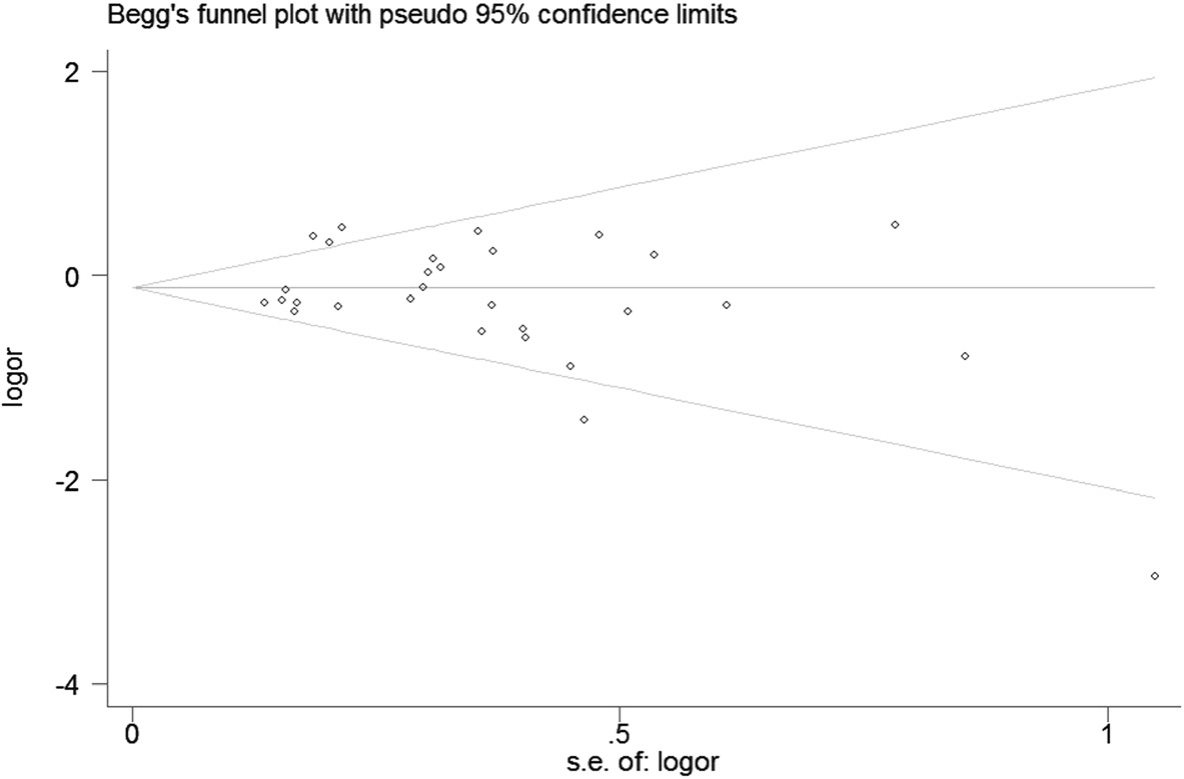
Begg funnel plot analysis to detect potential publication bias.

Begg’s funnel plot and Egger’s test were performed to assess the publication bias of the literature. The shapes of the funnel plots did not reveal any evidence of obvious asymmetry in all comparison models (Figure 4). Furthermore, Egger’s test was used to provide statistical evidence for funnel plot symmetry. The results still did not suggest any evidence of publication bias.

## Discussion

It is well recognized that there is a range of individual susceptibility to the same kind of cancer even with identical environmental exposure. Host factors, including polymorphisms of genes involved in carcinogenesis, may have accounted for this difference. Therefore, genetic susceptibility to cancer has been a research focus in scientific community. Among the common single nucleotide polymorphisms (SNPs) of the hOGG1 gene, one located in exon 7, resulting in an amino acid substitution of serine (Ser) with cysteine (Cys) at codon 326 (Ser326Cys, rs1052133), has been demonstrated to affect hOGG1 function [53]. The protein resulting from this substitution exhibits reduced DNA repair activity [53], and this SNP has been reported to be associated with the risk of many types of cancer [54]. Epidemiological studies of the OGG1 Ser326Cys polymorphism in relation to cancer have yielded mixed results with a weak association between the OGG1 Ser326Cys genotype and the risk of lung cancer. Wikman et al. carried out a case–control study which suggested that the hOGG1 polymorphisms were probably not major contributors to individual lung cancer susceptibility in Caucasians [33]. Hung et al. also observed that there were no such associations between them [36]. The same results were found in other studies [23]. Sugimura et al. found that the Ser326Cys polymorphism was not associated with an increased risk of lung cancer in any subtypes; however, when homozygous Cys326Cys were compared with other genotypes in combination, an increased risk was observed for the squamous cell carcinoma and nonadenocarcinoma after adjustment for age and smoking [32]. Ito et al. found no any effects of the OGG1 Ser326Cys polymorphism on the development of either adenocarcinomas or small cell carcinoma [34]. De Ruyck et al. found a reduced cancer risk associated with the OGG1 Ser326Cys polymorphism [40]. Individual studies on the relationships between SNPs of genes and cancer risk always yield inconsistent and controversial results partly because of a rather small sample size and low precision. Meta-analysis could solve the problem caused by the low statistical power of single studies and enable drawing of a more robust conclusion. Since there have been contradictory findings so far, we conducted a meta-analysis of 10,327 lung cancer cases and 12,148 controls to investigate its association with lung cancer risk.

The meta-analysis of Hung et al. showed that the summary OR was 1.37 (95% CI = 1.02–1.82) for the Cys/Cys genotype in various ethnic populations combined [55]. Li et al. performed another meta-analysis showed that individuals carrying the Cys/Cys genotype did not have significantly increased risk of lung cancer in all populations combined but, in the stratified analysis by ethnicity, a significantly increased risk was found among Asians (OR = 1.18, 95% CI = 1.01–1.38) [56]. Our work, including 22,475 subjects from 30 published case–control studies, explored the association between a potentially functional polymorphism, hOGG1 Ser326Cys and lung cancer susceptibility. We found that the variant genotypes of the hOGG1 were associated with a significant increased overall risk of lung cancer. When stratified according to ethnicity, Asians with the Ser/Ser showed a higher risk of lung cancer compared with those with the Cys/Cys genotype. However, Caucasians did not show the same risk. Ethnic difference in the association between lung cancer risk and the hOGG1 Ser326Cys polymorphism was suggested. Large studies including different ethnic groups with a careful matching between cases and controls should be considered in future association studies to confirm results from the meta-analyses.

Heterogeneity is a potential problem when interpreting the results of all meta-analyses. As looked through our study carefully, we found that the three studies [27,37,45] were noted to be a major source of heterogeneity. The reason may be that the study was only among non-smokers, and the controls were found to be out of HWE. Although this study was a major source of heterogeneity, the corresponding pooled ORs were not materially altered with or without including it almost in all genetic models, suggesting that the results of this meta-analysis are stable.

Some limitations of this meta-analysis should be addressed. Firstly, lung cancer is a multi-factorial disease that results from complex interactions between many genetic and environmental factors. This means that there will not be single gene or single environmental factor that has large effects on lung cancer susceptibility. Our results were based on unadjusted estimates, while a more precise analysis should be conducted if individual data were available, which would allow for the adjustment by other covariates including age, sex, family history, environmental factors and lifestyle. Secondly, in the subgroup analyses by ethnicity, control sources, the number of subjects was relatively small, not having enough statistical power to explore the real association. Thirdly, the controls were not uniformly defined. Although most of the controls were selected mainly from healthy populations, some had respiratory disease. Therefore, non-differential misclassification bias was possible because these studies may have included the control groups who had different risks of developing lung cancer.

## Conclusion

Despite some limitations listed above, this work suggests that the hOGG1 Ser326Cys variant is a risk factor for developing lung cancer. Additionally, we found that this phenomenon was more prominent in subgroups such as in Asians. However, it is necessary to conduct large studies using standardized unbiased methods, homogeneous lung cancer patients and well matched controls, with the assessors blinded to the data. Moreover, gene–gene and gene–environment interactions should also be considered in future analysis. Such studies taking these factors into account may eventually lead to our better, comprehensive understanding of the association between the hOGG1 Ser326Cys polymorphism and lung cancer risk.

## Competing interests

The authors declare that they have no competing interests.

## Authors’ contributions

ZX, LY, and XZ carried out the meta-analysis study, drafted the manuscript and involved in revising the manuscript critically for important intellectual content. ZX participated in the design of the study and revised the manuscript. ZX, LY, and XZ carried out the meta-analysis study and drafted the manuscript. ZX, LY, and XZ participated in the design of the study, drafted the manuscript and revised the manuscript. All authors read and approve the final manuscript.
